# Epidemiological surveillance of hand, foot and mouth disease in Shanghai in 2014–2016, prior to the introduction of the enterovirus 71 vaccine

**DOI:** 10.1038/s41426-018-0035-z

**Published:** 2018-03-21

**Authors:** Jingjing Li, Hao Pan, Xiangshi Wang, Qirong Zhu, Yanling Ge, Jiehao Cai, Yuefang Li, Aimei Xia, Jiayu Hu, Mei Zeng

**Affiliations:** 1 Department of Infectious Diseases, Children’s Hospital of Fudan University, Shanghai, 201102 China; 2 Department of Infectious Diseases and Control, Shanghai Municipal Center For Disease Control and Prevention, Shanghai, 200336 China

## Abstract

Hand, foot, and mouth disease (HFMD) is mainly epidemic in China and Southeast Asian countries. A novel enterovirus 71 vaccine has been available in China for preventing severe HFMD since 2016. Knowledge of the dynamic epidemiology of HFMD in different regions is necessary for appropriate intervention strategies. This study focused on the citywide surveillance data on the epidemiology and etiology of HFMD in Shanghai during 2014–2016. In these 3 years, the total numbers of reported HFMD cases were 65,018, 39,702, and 57,548, respectively; the numbers of severe cases (case-severity ratios) were 248 (0.38%), 35 (0.09%), and 59 (0.10%), respectively. Children <6 years old accounted for 86.65% to 89.34% of HFMD cases and 91.53 to 97.14% of severe cases. EV-A71 caused all three fatal cases. In severe cases, the detection rate of EV-A71 was 77.82% in 2014, 100% in 2015 and 98.31% in 2016. In uncomplicated inpatient cases, the detection rates of EV-A71, CV-A16, CV-A6, and CV-A10 were, respectively, 43.40, 22.10, 30.73, and 1.89% in 2014; 28.52, 6.46, 53.61, and 7.98% in 2015; and 31.79, 14.15, 44.55, and 4.64% in 2016. In mild community cases, the detection rates of EV-A71, CV-A16, CV-A6, and CV-A10 were, respectively, 25.78, 41.64, 22.93, and 1.78% in 2014; 17.41, 21.23, 50.99, and 3.15% in 2015; and 18.92, 27.84, 45.11, and 1.64% in 2016. Among the cluster outbreaks, the most common pathogen was CV-A16 in 2014 (50.69%) and 2015 (38.10%) and CV-A6 in 2016 (36.30%). These findings show that HFMD outbreaks remained at a high level in Shanghai during 2014–2016. CV-A6 was emerging as the most common pathogen causing HFMD.

## Introduction

Hand, foot and mouth disease (HFMD), caused by enteroviruses, is a common childhood infectious disease. Since the late 1990s, outbreaks of HFMD have been increasingly reported in the Asia-Pacific region. Enterovirus 71 (EV-A71) and coxsackievirus A16 (CV-A16) are the two major causative agents responsible^1^. In particular, EV-A71-associated outbreaks pose serious threats to public health due to the risk of severe neurological complications and mortality^2^.

HFMD has ranked first among the notifiable infectious diseases since 2010 in mainland China. As of 31 December 2016, a total of 14,609,859 cases, with 3058 fatal cases, were officially reported in mainland China^3,4^. In Shanghai, case series of HFMD were first reported in 1981^5^. Outbreaks of HFMD have been observed in Shanghai since 2007, in parallel with the national outbreak of HFMD^6,7^. EV-A71 and CV-A16 were responsible for 73% of HFMD cases in China between 2010 and 2012, and EV-A71 infections were associated with 93% of laboratory-confirmed deaths^8^. Consequently, EV-A71 vaccination was recommended to be included in the national expanded program of immunization of childhood since the EV-A71 vaccine was licensed in China in December 2015^9^. However, more recent studies are reporting a changing etiology of HFMD outbreaks in some regions of mainland China^10–14^. Hence, it is important to monitor the epidemiology and etiology of HFMD before the wide introduction of EV-A71 vaccination in Shanghai, which has the potential to achieve mass EV-A71 immunization in the near future. In this study, we characterized the epidemiology and predominant serotypes of HFMD in Shanghai from 2014 to 2016, aiming at informing the appropriate regional public health intervention strategies to prevent and control HFMD outbreaks.

## Results

### Demographic Characteristics of HFMD

Total HFMD case numbers of 65,018, 39,702, and 57,548, with 248, 35, and 59 severe cases, were reported in Shanghai in 2014, 2015, and 2016, respectively. The case-severity ratios were 0.38%, 0.09%, and 0.10%. Three patients died from cardio-pulmonary failure in 2014, but there were no fatalities in 2015 or 2016.

Males outnumbered females in each year, with the ratios of male to female being 1.46–1.50 in the total cases and 1.88–2.28 in the severe cases. Migrant case numbers outnumbered the local cases in 2014 (52.61% vs. 47.39%), while the local cases outnumbered the migrant cases in 2015 (54.22% vs. 45.78%) and in 2016 (54.66% vs. 45.34%). However, the majority of severe cases occurred in migrant children, comprising 73.79 to 85.71%.

Of the total number of HFMD patients, children aged <6 years accounted for 86.65 to 89.34%. Children aged 1–1.9 years accounted for the most cases each year (23.32 to 24.69%), followed by children 2–2.9 years in 2014 (19.70%) and children 3–3.9 years in 2015 and 2016 (19.22% and 18.99%, respectively; Table 1**)**. Children aged <1 year accounted for only 5.28 to 6.92%.

**Table 1:** The estimation of incidence and severity for HFMD of Shanghai in 2014–2016

	2014			2015			2016		
Items	Total cases	Severe cases	Case-severity ratio Per 100	Total cases	Severe cases	Case-severity ratio Per 100	Total cases	Severe cases	Case-severity ratio Per 100
Total cases	65,018	248	0.38	39,702	35	0.09	57,548	59	0.10
*Gender (n,%)*									
Male	38,766 (59.62%)	162(65.32%)		23,817 (59.98%)	23 (65.71%)		34,142 (59.33%)	41 (69.49%)	
Female	26,252 (40.38%)	86 (34.68%)		15,885 (40.02%)	12 (34.29%)		23,406 (40.67%)	18 (30.51%)	
Ratio	1.48	1.88		1.50	1.92		1.46	2.28	
*Household registration (n,%)*									
Local residents	30,810 (47.39%)	65 (26.21%)		21,525 (54.22%)	5 (14.29%)		31,454 (54.66%)	14 (23.73%)	
Migrant population	34,208 (52.61%)	183(73.79%)		18,177(45.78%)	30 (85.71%)		26,094 (45.34%)	45 (76.27%)	
Ratio	0.90	0.36		1.18	0.17		1.21	0.31	
*Age (n, %)*									
<1year	3431 (5.28%)	19 (7.66%)		2746 (6.92%)	3 (8.75%)		3498 (6.08%)	0	
1–1.9 years	15,159 (23.32%)	80 (32.26%)		9803 (24.69%)	7 (20.00%)		13,452 (23.37%)	14 (23.73%)	
2–2.9 years	12,808 (19.70%)	65 (26.21%)		7521 (18.94%)	4 (11.43%)		9792 (17.01%)	17 (28.81%)	
3–3.9 years	12,797 (19.68%)	33 (13.31%)		7629 (19.22%)	9 (25.71%)		10,931 (18.99%)	8 (13.56%)	
4–4.9 years	8589 (13.21%)	28 (11.29%)		4887 (12.31%)	6 (17.14%)		7888 (13.71%)	11 (18.64%)	
5–5.9 years	4930 (7.58%)	13 (5.24%)		2882 (7.26%)	5 (14.29%)		4306 (7.48%)	4 (6.78%)	
6–9.9 years	5690 (8.75%)	9 (3.63%)		1546 (3.89%)	0		5716 (9.93%)	5 (8.47%)	
10–14.9 years	522 (1.47%)	1 (0.39%)		1886 (4.75%)	1 (2.86%)		1365 (2.37%)	0	
≧ 15 years	558 (1.01%)	0		802 (2.02%)	0		605 (1.05%)	0	

Of the severe cases (age range: 3 months old to 14 years old), children <6 years accounted for 91.53 to 97.14%. The largest proportion of severe cases occurred in children aged 1–1.9 years in 2014 (32.26%); 3 ~ 3.9 years in 2015 (25.71%); and 2–2.9 years in 2016 (28.81%; Table 1).

### Seasonality of HFMD epidemics in Shanghai

**Fig. 1:**
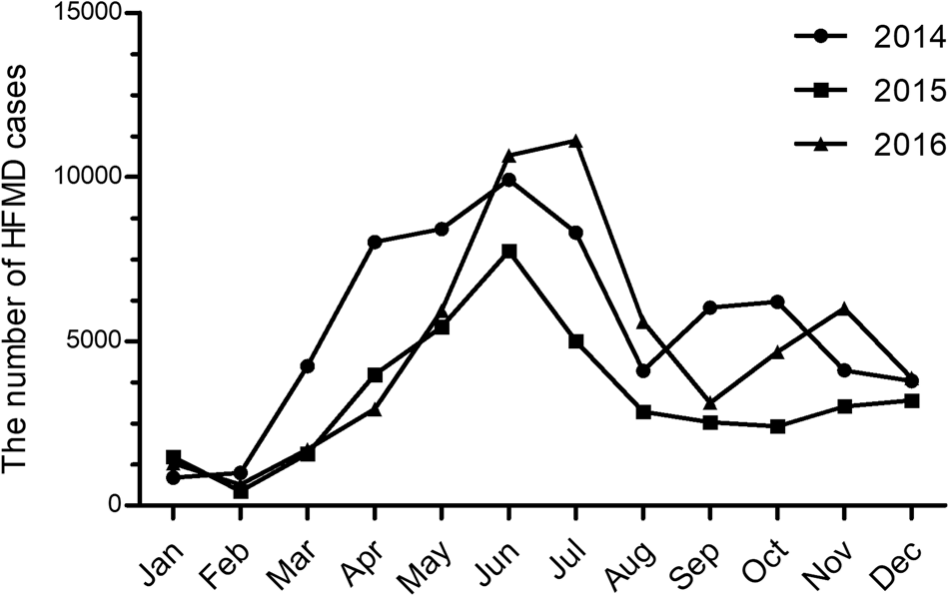
The monthly distribution of HFMD cases in Shanghai during 2014–2016

HFMD was prevalent throughout the year in Shanghai, but it peaked between April and July (late spring and summer) during 2014 and 2015 and between May and June in 2016. A second, smaller epidemic wave of HFMD was evident in the middle autumn or the early winter. This smaller epidemic appeared in September and October in 2014, in November and December in 2015, and in November in 2016. Overall, the intensity of the epidemics was significantly higher in 2014 and 2016 than in 2015 (Fig. 1).

### Enterovirus serotypes

Among the severe (community) HFMD cases, EV-A71 was detected in 77.82% of cases in 2014, 100% of cases in 2015, and 98.31% of cases in 2016; CV-A16 was detected in 1.67% of cases in 2015; and CV-A6 was detected in 1.69% of cases in 2016. All three fatal cases were laboratory confirmed to have EV-A71 infection.

Among the mild (community) HFMD cases, samples of which were monitored based on real-time RT-PCR in the CDC laboratory, CV-A16 was the most prevalent virus in 2014, with a detection rate of 41.64%, followed by EV-A71 (25.78%) and CV-A6 (22.93%). The detection rates of CV-A6, CV-A16 and EV-A71 were 50.99%, 17.41%, and 21.23%, respectively, in 2015 and 45.11%, 27.84% and 18.92%, respectively, in 2016. CV-A10 was detected less frequently, accounting for between 1.64 and 3.15% of cases, while other untyped enteroviruses accounted for between 6.49 and 7.87% of cases (Table 2).

**Table 2:** The etiology of HFMD by settings and disease severity in Shanghai during 2014–2016

Serotype	Mild cases in the community Number of cases (%)			Hospitalized cases without complications Number of cases (%)			Severe cases with meningitis/encephalitis Number of cases (%)			Cluster-associated HFMD outbreaks Number of episodes (%)		
	2014 (n* = 1513)	2015 (n* = 1206)	2016 (n* = 1401)	2014 (n* = 375)	2015 (n* = 263)	2016 (n* = 431)	2014 (n* = 239)	2015 (n* = 35)	2016 (n* = 59)	2014 (n* = 436)	2015 (n* = 231)	2016 (n* = 821)
EV-A71	390 (25.78%)	210 (17.41%)	265 (18.92%)	161 (42.93%)	75 (28.52%)	137 (31.79%)	186 (77.82%)	35 (100%)	58 (98.31%)	111 (25.45%)	44 (19.05%)	142 (17.30%)
CV-A16	630 (41.64%)	256 (21.23%)	390 (27.84%)	82 (21.87%)	17 (6.46%)	61 (14.15%)	4 (1.67%)	0 (0%)	0 (0%)	221 (50.69%%)	88 (38.10%)	271 (33.01%)
CV-A6	347 (22.93%)	615 (50.99%)	632 (45.11%)	114 (30.40%)	141 (53.61%)	192 (44.55%)	0 (0%)	0 (0%)	1 (1.69%)	53 (12.16%)	76 (32.90%)	298 (36.30%)
CV-A10	27 (1.78%)	38 (3.15%)	23 (1.64%)	7 (1.8%)	21 (7.98%)	20 (4.64%)	0 (0%)	0 (0%)	0 (0%)	0 (0%)	0 (0%)	24 (2.91%)
Pan-enterovirus	119 (7.87%)	87 (7.21%)	91 (6.49%)	11 (2.93%)	9 (3.42%)	21 (4.87%)	49 (20.67%)	0 (0%)	0 (0%)	51 (11.70%)	23 (9.95%)	86 (10.48%)

n* the number of cases or episodes sampled and tested positive for enterovirus.

Among uncomplicated HFMD inpatients whose samples were monitored based on nested RT-PCR and sequencing in the hospital research laboratory, EV-A71 was detected at a rate of 42.93%, followed by CV-A6 (30.40%), CV-A16 (21.87%), CV-A10 (1.80%), CV-A4 (1.07%), CV-A9 (0.8%), CV-A2 (0.53%), CV-A18 (0.27%) and Echovirus 18 (0.27%) in 2014; CV-A6 was detected at a rate of 53.61%, followed by EV-A71 (28.52%), CV-A10 (7.98%), CV-A16 (6.46%), CV-A2 (1.14%), CV-A4 (0.76%), Echovirus 30 (0.76%), CV-A18(0.38%), and Echovirus 18 (0.38%) in 2015; and CV-A6 was detected at a rate of 44.55%, followed by EV-A71 (31.79%), CV-A16 (14.15%), CV-A10 (4.64%), CV-A4 (3.25%), CV-A2 (0.70%), CV-B4 (0.23%), Echovirus 18 (0.23%), Echovirus 3 (0.23%) and Echovirus 30 (0.23%) in 2016 (Table 2).

During 2014–2016, the CDC notified the public about 1688, 1039, and 2675 cluster outbreaks, respectively, in kindergartens and schools. There were 436, 231, and 821 cluster outbreaks sampled in 2014, 2015, and 2016, respectively, for the identification of EV-A71, CV-A6, CV-A10, and CV-A16 based on real-time RT-PCR in the CDC laboratory. In 2014, EV-A71, CV-A16, CV-A6, and other enteroviruses were responsible for 25.45, 50.69, 12.16, and 11.70% of outbreaks, respectively; in 2015, EV-A71, CV-A16, CV-A6, and other enteroviruses were responsible for 19.05, 38.10, 32.90, and 9.95% of outbreaks, respectively; in 2016, EV-A71, CV-A16, CV-A6, CV-A10, and other enteroviruses were responsible for 17.30, 33.01, 36.30, 2.91, and 10.48% of outbreaks, respectively (Table 2). Meanwhile, 12, 4, and 5 outbreaks involving ≥10 cases of HFMD within 1 week in an institution were reported from 2014 to 2016; these outbreaks involved 242, 83, and 82 cases, respectively. Of those, 50, 28, and 31 cases were sampled for the detection of enterovirus. The detection rates of EV-A71, CV-A16, CV-A6, and other enteroviruses were 22.00, 48.00, 30.00, and 0%, respectively, in 2014; 25.00, 17.86, 42.86, and 3.57% in 2015; and 0, 35.48, 61.29, and 3.23% in 2016.

## Discussion

This study provides the latest epidemiological status of HFMD in Shanghai, the largest and most populous city in China. The intensity of HFMD outbreaks remained high during 2014–2016, with two annual peaks (one in the late spring and summer and one in the middle autumn and early winter). However, the overall case severity and fatality rates decreased remarkably compared to the surveillance data from 2007 to 2013 in Shanghai^6,15^. The reasons for the significant decline in the case-severity and the fatality rates are the replacement of EV-A71 predominance by non-EV-A71 serotypes in the community after 2014 and the implementation of aggressive interventions such as early recognition of cases at the community level, strict adherence to the national guidelines for management of HFMD/EV-A71 infection at the hospital level, and rigorous infection control in children’s institutions since 2011. In addition, this study reveals that CV-A6 replaced both EV-A71 and CV-A16 to become the most predominant enterovirus responsible for the community HFMD outbreaks in Shanghai since 2015. The replacement of the predominant serotype of enteroviruses presents a challenge for the prevention and control of outbreaks of HFMD in Shanghai and other regions in China in the post-EV-A71 vaccine era.

Based on the demographic characteristics of HFMD patients, boys were more frequently affected by HFMD and severe disease. This is consistent with prior Asian studies that showed that male sex was a risk factor for both mild and severe HFMD^16^. Strengthening the EV-A71 vaccination campaign among young boys is necessary. Overall, ~90% of HFMD cases occurred in children aged <6 years, with the greatest proportion of HFMD cases occurring in children aged 1–1.9 years. Infants were less frequently affected. Thus, EV-A71 vaccination among infants is recommended.

We noticed that severe cases occurred in children aged 3–4 years more frequently during 2015–2016 than during 2008–2014^6,15^. This age shift of severe cases from 1-year to 2-year-old children to preschool children suggests the necessity of EV-A71 vaccination among unvaccinated preschool children to prevent severe HFMD. Of note, the number of severe cases was 3–6 times greater among migrant children than among local children during 2015–2016, although the number of mild cases was greater among local children than among migrant children.

In 2007, when HFMD became epidemic in Shanghai, we observed that the initial outbreak of HFMD started among local institutionalized preschool children^6^; subsequently, migrant cases outnumbered local cases, with home-cared young children affected most commonly during 2008–2014^6^. The observed changing trend of demographic characteristics during 2015–2016 could be explained by the significant shift of the serotype of enteroviruses causing HFMD in Shanghai and the accumulation of susceptible local children. It is possible that local Shanghainese children became more susceptible because they lacked protective immunity against the current predominant serotypes, especially CV-A6.

EV-A71 vaccine development was listed as one of the national priorities following the 2008 national outbreak. A previous Shanghai study revealed EV-A71 as the predominant causative agent for HFMD in Shanghai from 2009 to 2011, accounting for 33.30%, 85.42%, and 93.75% of mild, severe, and fatal cases, respectively, and 41.76% of uncomplicated hospitalized cases^6,17,18^. In theory, mass EV-A71 vaccination will be cost-effective and useful for reducing the disease burden of HFMD and preventing severe HFMD if EV-A71 remains the predominant pathogen responsible for HFMD in Shanghai and across the country^19,20^. However, the predominance of EV-A71 has decreased markedly since 2013 in Shanghai: the 2013 surveillance data from the Shanghai CDC show that EV-A71 and CV-A16 were no longer the most common pathogens responsible for HFMD in the community, with detection rates of 16.78% and 8.36%, respectively. Non-EV-A71 and non-CV-A16 serotypes accounted for 74.86%^15^. In 2013, CV-A6 emerged as the most common cause of HFMD in Guangdong, Fujian, Beijing, Changchun, and Suzhou, with detection rates of between 39.6 and 66.9%, much higher than the detection rates of EV-A71 (14.6%–40.23%)^10–12,21–23^. This study shows that EV-A71 was no longer the dominant serotype causing HFMD in either the community setting or the outbreak-associated setting, in contrast to the situation in Shanghai in 2011^24^. CV-A6 became predominant over EV-A71 and second only to CV-A16 in Shanghai in the community in 2014 and surpassed both EV-A71 and CV-A16 as the most common serotype causing HFMD during 2015–2016. In addition, CV-A16 remained the most common serotype associated with HFMD in mild cases during 2014–2016. A recent study conducted in France showed that CV-A6 was also the most frequent serotype involved in HFMD outbreaks during 2014–2015, with a detection rate of 53.9%^25^. As early as 2008, CV-A6 caused a HFMD outbreak in Finland and then circulated in France, Spain, and other European countries from 2009 to 2011^26-28^. In Asia, CV-A6 was responsible for HFMD outbreaks in Singapore in 2009, Taiwan in 2009–2010, Japan in 2011, and Thailand in 2012^29-32^. In the USA, CV-A6-associated HFMD outbreaks were reported from November 2011 to February 2012^33^. A Shanghai study detected CV-A6 and CV-A10 at only very low frequencies during 2008–2009^34^. Nevertheless, taken together, the data suggest that CV-A6 is becoming an important cause of outbreaks of HFMD in mainland China and worldwide.

Although CV-A10 was an uncommon serotype in HFMD outbreaks in Shanghai during 2014–2016, the possible upsurge of CV-A10 in the next few years requires attention. CV-A10 was reported to cause outbreaks of HFMD in Wuhan, with a detection rate as high as 41.04%, followed by EV-A71 (21.2%), during 2012–2013^35^. CV-A10 infection also led to fatal HFMD due to cardio-respiratory failure in Henan province and severe HFMD in Qingdao^36,37^.

In 2016, EV-A71 vaccines became available on the Chinese market. EV-A71 vaccination was recommended for children aged between 6 and 71 months. Many regional governments just finished the tender process for EV-A71 vaccines by 2016, and EV-A71 vaccination has been undertaken nationally since the end of 2017. Before and after EV-A71 vaccination becomes widely implemented in China, monitoring of the serotypes associated with HFMD outbreaks, especially EV-A71, CV-A6, CV-A16, and CV-A10, is vital for the early identification of emerging enteroviruses and for the evaluation of the HFMD-protective effect of EV-A71 vaccination and its impact on the public health burden of this disease in our country. As a re-emerging pathogen of HFMD epidemics, CV-A6 has become a significant challenge in the control of HFMD. Monitoring of the CV-A6 and CA10 epidemics and development of a multivalent vaccine against CV-A6, EV-A71, and CV-A16 are needed in the near future.

## Materials and methods

### HFMD surveillance and case definition in Shanghai

HFMD has been listed as a notifiable infectious disease since 2007. For this reason, local health providers and physicians are required to report clinically diagnosed HFMD cases to the Shanghai Municipal Center for Disease Control and Prevention (CDC) within 24 h via an internet-based surveillance system. Epidemiologic and clinical information was recorded for each HFMD patient. Daycare centers, kindergartens, and schools report HFMD clusters/outbreaks to the CDC. A cluster outbreak is defined as ≥2 cases of HFMD occurring in a classroom within 1 week or ≥5 cases of HFMD occurring in an institution within 1 week. An outbreak is defined as ≥10 cases of HFMD occurring within 1 week in an institution. If ≥10 cases of HFMD occur in a span of more than 1 week in an institution, such a situation is defined as a cluster.

An HFMD case is characterized by oral vesicular exanthema/ulcers with vesicular lesions on the hands, feet, and/or buttocks. Cases were classified as mild (community) cases, inpatient cases without complications, and severe inpatient cases with complications. A severe case was defined as HFMD accompanied by at least 1 of the following complications: aseptic meningitis, encephalitis, acute flaccid paralysis, pulmonary edema or hemorrhage, or cardiopulmonary collapse. Cerebrospinal fluid pleocytosis was defined as a white blood cell count more than 10 × 10^6^ cell/L in a patient >1 month of age.

### Laboratory-based surveillance for enterovirus

In Shanghai, all the 18-district CDC laboratories perform routine citywide surveillance of enterovirus among mild (community) HFMD cases, severe HFMD cases, cluster-associated HFMD cases, and outbreak-associated HFMD cases. Stool samples or rectal swabs and throat swabs are collected by physicians for enterovirus detection. The protocols for enterovirus surveillance were as follows. For mild cases, the first 5–10 outpatients with notifiable mild HFMD in each district of Shanghai were enrolled per month; for severe cases, all confirmed patients were enrolled; for a cluster of ≥5 HFMD cases occurring in an institution, 2 patients were sampled; for an outbreak of ≥10 HFMD cases occurring within 1 week in an institution, 5 patients were sampled.

Routine enterovirus surveillance among hospitalized HFMD patients was performed at the Children’s Hospital of Fudan University, to which ~80% of HFMD inpatients and 95% of patients with severe cases reported in Shanghai are admitted annually. Stool samples taken from 50 to 75% of HFMD inpatients were used for enterovirus surveillance.

### Enterovirus detection and serotyping

The stools, rectal swabs, and throat swabs sent to the CDC laboratories were stored in MEM (minimum essential medium) at −20 °C. Virus RNA was extracted using a MagNA Pure LC Total Nucleic Acid Isolation Kit–Large Volume (ROCHE, Co, USA) according to the manufacturer’s instructions. The CDC laboratories used commercial one-step real-time RT-PCR kits (Da An GeneCo, Ltd., Guangzhou, China or Jiangsu Bioperfectus Technologies Co, Ltd, China) to detect pan-enterovirus, EV-A71, CV-A16, CV-A6, and CV-A10 in the specimens collected for routine surveillance. The detection sensitivity of the kit was 1 × 10^3^ PFU/mL, and a specimen was considered positive for virus if reaction growth curves crossed the threshold line within 35.1 cycles, according to the manufacturer’s instructions.

The stool samples sent to the hospital research laboratory were stored at −20 °C and thawed for preparation of 20% stool suspension in sterile normal saline prior to RNA extraction. Viral RNA was extracted using a TIANamp Virus RNA Mini Kit (Tiangen Biotech, Beijing, China) according to the manufacturer’s protocol. The hospital laboratory used above mentioned commercial one-step real-time RT-PCR kits (Da An GeneCo., Ltd. Guangzhou, China or Jiangsu Bioperfectus Technologies Co, Ltd, China) to detect pan-enterovirus, EV-A71 and CV-A16. A pan-enterovirus-specific primer targeting the 5′UTR for nested RT-PCR, to give a 389-bp product, was used to identify the serotypes of enteroviruses in stool specimens negative for EV-A71 and CV-A16. The pan-enterovirus-specific primers for the 5′UTR were described previously by Ge et al.^38^ Gel-purified PCR products were bi-directionally sequenced using an ABI 3730xl automatic DNA analyzer (Meiji Gene Company, Shanghai, China). The sequences were then analyzed using the BLAST program against sequences available in the GenBank database to identify the serotype.

### Statistical analysis

The case-severity rate was defined as the proportion of severe disease among all reported cases, the case-fatality rate was defined as the proportion of fatal cases among all reported cases, and the severe case-fatality rate was defined as the proportion of fatal cases among all severe cases. The data were analyzed using SPSS version 13.0 (SPSS Inc., Chicago, IL) software.
